# Exploring the Influence of Dysphagia and Tracheostomy on Pneumonia in Patients with Stroke: A Retrospective Cohort Study

**DOI:** 10.3390/brainsci12121664

**Published:** 2022-12-03

**Authors:** Yong Dai, Jia Qiao, Qiu-Ping Ye, Xin-Ya Li, Jia-Hui Hu, Zu-Lin Dou

**Affiliations:** 1 Clinical Medical College of Acupuncture-Moxibustion and Rehabilitation, Guangzhou University of Chinese Medicine, Guangzhou 510006, China; 2 Department of Rehabilitation Medicine, the Third Affiliated Hospital of Sun Yat-sen University, Guangzhou 510630, China

**Keywords:** tracheostomy, stroke, pneumonia, dysphagia, retrospective study

## Abstract

Background: Pneumonia is common in patients with tracheostomy and dysphagia. However, the influence of dysphagia and tracheostomy on pneumonia in patients with stroke remains unclear. The aim of this study was to explore the risk factors related to pneumonia, and the association between dysphagia, tracheostomy and pneumonia in patients with stroke was investigated. Methods: Patients with stroke who experienced tracheostomy and dysphagia were included and divided into two groups based on record of pneumonia at discharge. Clinical manifestations and physical examination were used to diagnose pneumonia, whereas clinical swallowing examination, and videofluoroscopy swallowing studies (VFSS) were used to evaluate swallowing function. Results: There were significant differences between the pneumonia group and the no pneumonia group in total tracheostomy time (6.3 ± 5.9 vs. 4.3 ± 1.7 months, *p* = 0.003), number of instances of ventilator support (0.41 ± 0.49 vs. 0.18 ± 0.38, *p* = 0.007), PAS score (5.2 ± 1.92 vs. 4.3 ± 1.79, *p* = 0.039), impaired or absent cough reflex (76.4 vs. 55.6%, *p* = 0.035), oropharyngeal phase dysfunction (60.6 vs. 40.8%, *p* = 0.047), length of hospital stay (36.0 ± 7.2 vs. 30.5 ± 11.7 days, *p* = 0.025) and direct medical costs (15,702.21 ± 14,244.61 vs. 10,923.99 ± 7250.14 United States dollar [USD], *p* = 0.042). Multivariate logistic regression showed that the total tracheostomy time (95% confidence interval [CI], 1.966–12.922, *p* = 0.001), impaired or absent cough reflex (95% CI, 0.084–0.695, *p* = 0.008), and oropharyngeal phase dysfunction (95% CI, 1.087–8.148, *p* = 0.034) were risk factors for pneumonia. Spearman’s correlation analysis demonstrated that PAS scores were significantly correlated with cough reflex dysfunction (r = 0.277, *p* = 0.03), oropharyngeal phase dysfunction (r = 0.318, *p* < 0.01) and total tracheostomy time (r = 0.178, *p* = 0.045). The oropharyngeal phase dysfunction was significantly correlated with cough reflex (r = 0.549, *p* < 0.001) and UES opening (r = 0.643, *p* < 0.01). Conclusions: Tracheostomy and dysphagia increased the risk of pneumonia in patients with stroke. Total tracheostomy time, duration of ventilator support, degree of penetration and aspiration, and oropharyngeal phase dysfunction are risk factors. Given this, we also found that there may be a correlation between tracheostomy and dysphagia.

## 1. Introduction

Stroke is the second leading cause of death and the third leading cause of disability worldwide, and the burden of this disease is rapidly increasing in low- and middle-income countries [1,2]. Tracheostomy is a vital rescue intervention for patients with stroke who have reduced airway protection reflexes, and thus high risk of aspiration and/or depressed consciousness [3]. It is a surgical procedure that involves cutting into the trachea and inserting a tube into the opening. These artificial airways provide direct, unobstructed lower respiratory tract access to maximize ventilation, expedite oxygen entry, and facilitate secretion management [4]. Despite its benefits, tracheostomy often co-exists with dysphagia (11% to 93%) [5,6], and is associated with adverse medical outcomes, most notably increased risk of pneumonia and malnutrition [7]. In addition, patients with dysphagia and pneumonia are more likely to develop worse functional outcomes, higher mortality, and increased readmissions and institutionalization [8].

Previously, aspiration after tracheostomy has been reported in 50–87% of patients [9], and the subglottic secretions above the endotracheal cuff are associated with bacteria colonization of lower respiratory tract, resulting in pneumonia [10]. There are several posited explanations for why pneumonia occurs in patients with tracheostomy and dysphagia, including decreases in the strength of sensory input, disuse atrophy of laryngeal structures, and reduced subglottic air pressure [11]; however, this risk does not appear to be widely recognized [12] as the relationship between tracheostomy and pneumonia is controversial, and inflated endotracheal tube cuffs to prevent aspiration of water into the lung have been reported [8]. Currently, the risks factors related to pneumonia in patients with dysphagia and tracheostomy remains unclear [13,14], and the relationships among tracheostomy, dysphagia and pneumonia in patients with stroke have not been well described [15,16]. Therefore, the current study aimed to explore the influence of dysphagia and tracheostomy on pneumonia in patients with stroke, and to clarify possible association between dysphagia, tracheostomy and pneumonia.

## 2. Materials and Methods

### 2.1. Study Design and Subjects

This was a retrospective study; the data were extracted from the cohort database of the Third Affiliated Hospital of Sun Yat-sen University from January 2010 to June 2022. Stroke patients who had undergone tracheostomy were identified, and detailed clinical information was collated, including medical records, auxiliary examinations, and treatment processes. A standardized identified data collection form was used to collect data from the medical records. Collected data included: demographic details (age, sex); stroke type; stroke location; comorbidities (coronary heart disease, hypertension, diabetes, hyperlipidemia); personal history (smoking and drinking); videofluoroscopy swallowing study (VFSS) assessment results, including the penetration–aspiration scale (PAS), upper esophageal sphincter (UES) opening, cough reflex, and swallowing phase; total tracheostomy time (the duration of tracheal cannula), number of instances of ventilator support, hospital length of stay, and direct medical costs. Dysphagia was confirmed by specifically-trained speech–language therapists specializing in post-stroke dysphagia based on swallowing screening, detailed clinical swallowing evaluation, and VFSS examination. Ethical approval was obtained from the ethical committees of the Third Affiliated Hospital of Sun Yat-sen University (02-351-01). 

### 2.2. Inclusion and Exclusion Criteria

The inclusion criteria were age > 18 years; stroke was diagnosed and confirmed by computed tomography and/or magnetic resonance imaging of the brain, performed at the acute stage in all patients (hemorrhagic or ischemic stroke, such as subdural hematoma, subarachnoid hemorrhage, intracerebral hemorrhage, and ischemia); tracheostomy performed after stroke; swallowing function evaluation by speech language therapists at admission to the rehabilitation department; and pneumonia diagnosed during hospitalization after tracheostomy. The exclusion criteria were incomplete case information; neurological diseases other than stroke (e.g., brain tumor, Parkinson’s disease, neuromyelitis optica); pre-existing diseases causing dysphagia (e.g., structural abnormalities of the mouth or throat, esophageal cancer); diagnosis of pneumonia at admission; high-risk diseases such as chronic obstructive pulmonary disease, lung transplantation, immune system dysfunction, respiratory disease; severe cognitive impairment, including delirium, dementia, coma, vegetative state; and other surgeries such as neck surgery, lung transplantation, thoracotomy, chemotherapy for nasopharyngeal cancer, and tongue cancer.

### 2.3. Evaluation Methods

#### 2.3.1. Pneumonia Diagnosis

Pneumonia is identified using a combination of imaging, clinical and laboratory criteria. In the current study, the diagnostic criteria for pneumonia were as follows: (A) The relevant clinical characteristics of pneumonia: new onset of cough or exacerbation of symptoms of existing respiratory diseases, with or without purulent sputum, chest pain, dyspnea, or hemoptysis; fever; evidence of pulmonary consolidation and/or moist rales; Peripheral white blood cell count (WBC) > 10 × 10^9^/L or <4 × 10^9^/L, with or without a left shift. (B) The chest radiograph showing new patchy infiltrates, ground-glass opacities, or interstitial changes, lobar or segmental consolidation, with or without pleural effusion [17]. Clinical diagnosis was established if a patient satisfied criterion B and any one condition of criterion A. Patients with tuberculosis, pulmonary tumor, non-infectious interstitial lung disease, pulmonary edema, atelectasis, pulmonary embolism, pulmonary eosinophilia, and pulmonary vasculitis were excluded [18]. 

#### 2.3.2. Swallowing Function Evaluation

All tracheostomy patients underwent clinical swallowing evaluations at admission to the rehabilitation department. Typical components included dysphagia specific anamnesis, dysphagia screening and examination, and instrumental examination methods [19]. The underlying diseases, comorbidities, drug history, previous diagnosis and therapeutic trials and dysphagia specific issues were also evaluated [20], and, in particular, there was a specific need to ask about the occurrence of pneumonia.

The volume–viscosity swallow test (V-VST) was used to detect patients’ signs of impaired safety and efficacy of swallow [21]. Patients start the test with a 5 mL medium bolus, followed by 10 and 20 mL, then perform with low viscosity boluses following the same volumetric approach, and finally complete the test with high viscosity boluses. The V-VST should be ended if any safety impairment with high viscosity happened [22]. If the V-VST is positive, tracheostomized patients whose neurological and vital signs were stable undertook the VFSS examination at admission to the rehabilitation department with specifically trained speech-language therapists [23]. The first record was included in the analysis. The PAS score, UES opening, cough reflex, and swallowing phase dysfunction obtained from VFSS were adopted. Videofluoroscopy was performed using a fluoroscopy unit (Siemens ICONOS R200, Siemens AG, Erlangen, Germany, 2005) with an image rate of 30 pulses/second. The procedure was performed according to the modified Logemann protocol [24]. 

PAS is an 8-point scale used to evaluate the severity of penetration and aspiration. The higher the score, the more severe the penetration and aspiration symptoms [25]. Complete airway protection was given a PAS score of 1, while the impermanent entry of the bolus into the laryngeal vestibule (above the vocal folds) was given a score of 2. If the bolus was not cleared from the laryngeal vestibule the score was 3–5 according to the depth and amount. Scores of 6–8 were given in the case of aspiration, when the bolus crossed the vocal folds into the trachea. In cases with multiple sub-swallows in one swallowing trial, swallowing safety was recorded based on the worst score obtained on the PAS. Swallowing dysfunction in the oral, pharyngeal, and esophageal phases were recorded. The definitions of the different phases are shown in Table 1 [26]. Different measures were analyzed using Image J open-source software (National Institutes of Health, Bethesda, Maryland) by specifically trained speech-language therapists with more than two years of work experience. During the analysis, the video was played at a speed of 30 frames/s and played back frame-by-frame; each video image was rated in duplicate by two trained raters, and any discrepancies across ratings were resolved through consensus with a third experienced rater [27].

### 2.4. Statistical Analysis

Continuous variables were tested for normality using the Kolmogorov–Smirnov test. The Student’s *t*-test was used for continuous data and Fisher’s exact test for categorical data. Differences between groups were determined using Pearson’s chi-squared test (χ2) or Fisher’s exact test (as appropriate). PAS was analyzed using the Wilcoxon rank sum test in the prespecified subgroup analysis. Risk factors for pneumonia were investigated using multivariate logistic regression. The correlation between PAS, UES opening, impaired or absent cough reflex, oropharyngeal phase dysfunction, total tracheostomy time, and number of instances of ventilator support was analyzed using the Spearman correlation analysis. All comparisons were two-tailed. The statistical significance level was set at *p* < 0.05. All statistical analyses were performed using IBM SPSS Statistics for Windows, version 23 (IBM Corp., Armonk, NY, USA, 2015).

## 3. Results

**Figure 1:**
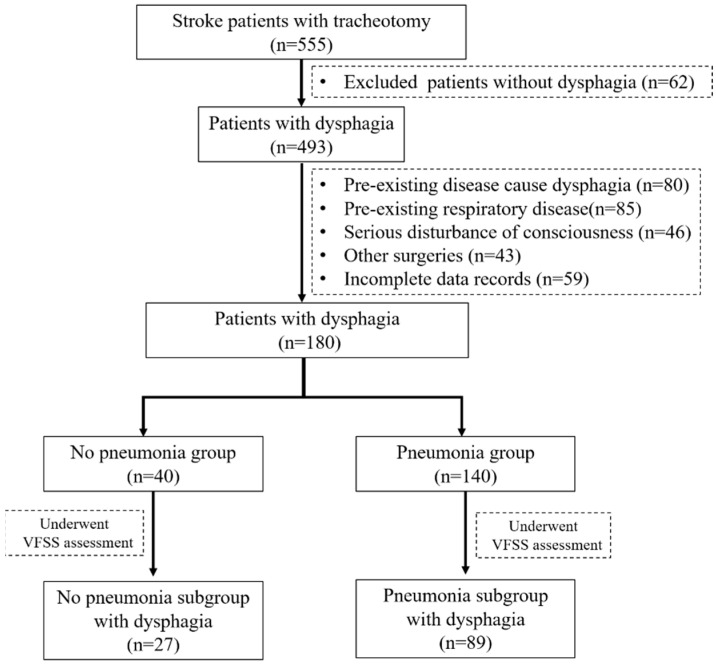
The flow chart of the study design.

A total of 555 patients with tracheostomy after stroke were screened, of which 180 patients diagnosed with dysphagia were included in the analysis of risk factors for pneumonia, including 140 patients in the pneumonia group and 40 in the no pneumonia group. In this study, dysphagia was diagnosed based on swallowing function assessment, including clinical symptoms, FOIS, V-VST, or VFSS. To further explore the relationship between dysphagia and pneumonia, only 116 patients who underwent VFSS were included in the subgroup analysis (Figure 1).

### 3.1. The Influence of Tracheostomy on Pneumonia

Among 180 patients with dysphagia, 140 were in the pneumonia group, and 40 were in the no pneumonia group. There were no significant differences in terms of sex, age, stroke type, stroke location, feeding behavior, and history of disease between the two groups (*p* > 0.05) (Table 2). However, the comparative analysis demonstrated that the total tracheostomy time for patients in the pneumonia group (6.3 ± 5.9 months) was significantly longer than for patients in the no pneumonia group (4.3 ± 1.7 months) (*p* = 0.037). A significant difference was found in the number of instances of ventilator support between the pneumonia (0.41 ± 0.49) and the no pneumonia groups (0.18 ± 0.38) (*p* = 0.007). In addition, a longer length of hospital stays and higher direct medical costs during hospitalization were observed in the pneumonia group (36.0 ± 7.2 days; 15,702.21 ± 14,244.61 USD, respectively) than in the no pneumonia group (30.5 ± 11.7 days; 10,923.99 ± 7250.14 USD, respectively) (*p* = 0.025; *p* = 0.042, respectively). 

### 3.2. Subgroup Analysis of the Influence of Dysphagia on Pneumonia

Finally, 89 and 27 patients in the pneumonia and no pneumonia group, respectively, were included to explore the relationship between dysphagia and pneumonia. A significant difference in PAS scores was found between the pneumonia (5.2 ± 1.92) and the no pneumonia group (4.3 ± 1.79) (*p* = 0.039) (Table 3). Impaired/absent cough reflex was more common in the pneumonia (76.4%) compared with the no pneumonia group (55.6%) (*p* = 0.035) (Table 3). However, no difference was found between the groups in UES opening (Table 3). Furthermore, there were statistical differences in oropharyngeal phase dysfunction between the pneumonia and no pneumonia subgroups (*p* = 0.047), however, no differences were found for other phases of swallowing (Table 4). The post hoc testing of differences in the phase of swallowing between groups are showed in Table 5. 

### 3.3. The Risk Factors of Pneumonia

There were significant differences in total tracheostomy time, number of instances of ventilator support, PAS score, cough reflex, and oropharyngeal phase dysphagia through descriptive statistics (*p* < 0.05, Table 2). When these factors were included in the logistic regression analysis, the results suggested that total tracheostomy time; (odds ratio [OR] = 5.040; *p* = 0.001), cough reflex dysfunction (OR = 0.241; *p* = 0.008), and oropharyngeal phase dysfunction (OR = 2.976; *p* = 0.034) were risk factors for pneumonia in patients with stroke, dysphagia and tracheostomy (Table 6).

### 3.4. Correlation Analysis of Tracheostomy and Dysphagia

Spearman’s correlation analysis was performed for PAS scores, UES opening, cough reflex, oropharyngeal phase dysfunction, total tracheostomy time, and number of instances of ventilator support. PAS scores were significantly correlated with cough reflex dysfunction (r = 0.277, *p* = 0.03), oropharyngeal phase dysfunction (r = 0.318, *p* < 0.01) and total tracheostomy time (r = 0.178, *p* = 0.045). The oropharyngeal phase dysfunction was significantly correlated with cough reflex (r = 0.549, *p* < 0.001) and UES opening (r = 0.643, *p* < 0.01) (Table 7).

## 4. Discussion

The present study suggests that tracheostomy and dysphagia might increase the incidence of pneumonia in patients with stroke. In addition, the total tracheostomy time, impaired or absent cough reflex dysfunction, and oropharyngeal phase dysfunction are risk factors for pneumonia. Furthermore, there may be a correlation among PAS score, total tracheostomy time, cough reflex, UES opening and oropharyngeal phase dysfunction. Lastly, patients with pneumonia may have an increased length of hospital stay and higher direct medical costs.

We found that patients with tracheostomy, with a longer tracheostomy time and more ventilator support, were more likely to develop pneumonia. Tracheostomy allows air to enter the lungs, improving oxygenation and ventilation effectively. However, it is a double-edged sword, as when breathing occurs through the tube, bypassing from the mouth, nose, and throat, the risk of aspiration and pneumonia may increase [28,29]. There were several possible causative mechanisms for pneumonia in patients with tracheostomy. First of all, tracheostomy leads to pathophysiological changes in the upper airway and pharyngeal cavity, change in airway resistance and a decrease in pharyngeal pressure during swallowing, especially the subglottic air pressure [30]. In addition, tracheostomy bypasses upper airway defense mechanisms, such as upper airway humidification and ciliary movement, causing desensitization of the larynx and loss of the protective reflex due to chronic air diversion. Furthermore, the coordination between swallowing and breathing is impaired in patients with a tracheostomy [31]. Studies have confirmed that swallowing and breathing are coordinated, which is considered to be one of the mechanisms of airway protection [32]; additionally, tube cuff compression can result in vocal cord paresis/paralysis and may prohibit competent airway protection, resulting in insufficient airway closure, reduced cough reflex, and decreased airway protection, which may increase the risk of pneumonia [14].

Although several possible causative mechanisms have been suggested, the association of tracheostomy with an increased incidence of aspiration remains under debate. Some other experts and scholars believe that a tracheal cannula with an inflated cuff protects from aspiration, and is thus assumed to lower the risk of pneumonia in these patients [12,13]. The results of this study may serve as supporting evidence. The longer the duration of endotracheal intubation and tracheostomy time, the greater the damage to airway integrity. Most studies have reported total tracheostomy time and the benefits of its reduction [33]. Christopher et al. demonstrated that earlier decannulation has important clinical benefits for speech and swallowing, and restoring normal respiratory physiology including cough function [34]. There is also less risk for adverse events and reduced potential for infection [35]. Moreover, patients with more ventilator support are more likely to suffer from pneumonia. This may be related to respiratory muscle weakness. Symptoms of respiratory muscle weakness are common in patients with mechanical ventilation and long-term intubation, which cause a significant decrease in the ability to cough and clear secretions, which can develop into pneumonia. Membrane proteolysis can be detected in patients with invasive mechanical ventilation within 18 to 69 hours. This means that the respiratory muscles rapidly atrophy [36]. After 24 hours of mechanical ventilation, respiratory muscle weakness was almost twice as high as limb muscle weakness (63% vs. 34%) [37].

The stage of dysphagia may be related to stroke and tracheostomy. Chang et al. reported that in patients with acute cerebral infarction, dysphagia usually occurs in the oral and pharyngeal stages [38]. Betts et al. reported that the tube cuff could obstruct the pharyngeal pathway directly [39]. After tracheostomy, the mechanical effects of a tracheostomy tube may negatively affect swallowing function, including a reduction in laryngeal elevation, less subglottic pressure, reduced vocal fold adduction reflex, increased external cuff pressure in the esophagus and occurrence of stasis in the supraglottic region, While the function of food transport in the oral cavity and pharynx is impaired, oropharyngeal dysphagia occurs, increasing the risk of aspiration [40,41]. These findings were similar to those in our study; tracheostomized patients after stroke accompanied with pneumonia tend to manifest oropharyngeal phase dysfunction.

However, recent studies have indicated that the presence of a tracheostomy tube may not affect the biomechanics or kinematics of swallowing. Terk et al. reported that tracheostomy did not significantly alter hyoid bone movement and laryngeal excursion, which are important components of normal pharyngeal swallow biomechanics [42]. Kang et al., also found that the extubation of a tracheostomy tube did not affect the kinematics of swallowing, implying no relationship between tracheostomy tube placement and dysphagia [11]. More recently, Park et al. reported that swallowing function did not change before and after tracheostomy decannulation [40]. In our study, a correlation was observed between tracheostomy time and PAS score (r = 0.178, *p* = 0.045), and no correlation was found in other indicators, such as oropharyngeal phase dysfunction and UES opening, which may suggest that dysphagia is related to tracheostomy but there is insufficient evidence. Hence, future study will be needed to verify the relationship between tracheostomy and dysphagia.

In addition, a significant correlation between dysphagia and pneumonia was found. Patients with higher PAS scores and impaired cough reflex were more likely to develop pneumonia, and cough reflex was significantly correlated with PAS score (r = 0.277, *p* = 0.03). Coughing is one of the defense mechanisms that can effectively prevent foreign bodies from entering the airway and lungs, and reduce the risks of aspiration. However, tracheostomized patients often have difficulty in initiating the compressive coughing phase, and cough flows are typically insufficient [43]. McKim et al. demonstrated that peak cough flow significantly increased after tracheostomy tube decannulation [44].

Moreover, oropharyngeal phase dysfunction was significantly correlated with cough reflex (r = 0.549, *p* < 0.001) and UES opening (r = 0.643, *p* < 0.001), as well as PAS score (r = 0.318, *p* < 0.001). Oropharyngeal dysphagia is a health problem that refers to a disturbance in the oral preparatory, oral, and/or pharyngeal phases of swallowing, resulting in difficulty in eating, drinking, or swallowing. Oropharyngeal aspiration causes frequent respiratory infections and aspiration pneumonias. Due to the complex pathophysiology and great differences in etiologies and manifestations, the diagnosis of oropharyngeal dysphagia remains a challenge [45]. In our study, cough reflex, UES opening, and PAS score were all related to oropharyngeal phase dysfunction. Therefore, current research aimed at improving the diagnosis and treatment of oropharyngeal dysphagia still needs to be further carried out in patients with stroke and tracheostomy.

We found that males were over-represented in this cohort. This may be related to the epidemiological characteristics of stroke and tracheostomy. A previous review article focusing on the sex-specific information showed that male stroke incidence rate was 33% higher than for females [46]. In recent years, genetic factors, the positive effects of estrogen on cerebral circulation, and blood pressure were considered as the epidemiological differences between the sexes [47]. There is no specific report on the relationship between tracheostomy and gender, but in previous retrospective analyses with large sample sizes, the risk of tracheostomy complications in male patients was higher than that in female patients [48]. In summary, the ratio of males in this study is similar to that in previous studies, which may suggest that males are at higher risk of developing stroke and tracheostomy, but the specific relationship needs to be studied with a larger sample size.

Patients with pneumonia after tracheostomy experienced a longer length of hospital stay and higher direct medical costs than those without pneumonia. In the present study, it means that the burden of pneumonia in patients after stroke, accompanied with tracheostomy and dysphagia, may be increased; more medical resources are needed for patients with pneumonia after stroke, such as airway management, drugs, and physiotherapy. Some studies indicate that pneumonia may be a risk factor that increases the burden of tracheostomy and stroke [49,50]. More recently, Kaur et al. reviewed the costs of stroke in low- and middle-income countries, mostly in Asia. The average length of hospital stay was longest (20 days) in China [51], which was a significantly lower hospital stay than in this study. Meanwhile, there are many evidence-based guidelines for tracheostomy management reporting that multidisciplinary methods have benefits for reducing pneumonia risk, shortening the length of hospital stay and saving costs [7]. We may be able to conduct research on this in a future study.

The current study had several limitations. First, only 116 patients who had complete VFSS medical records were included in the subgroup analysis to explore the relationship between dysphagia and pneumonia. Moreover, we lacked a comparison with patients who did not have dysphagia. Some parameters are lacking due to the limitations of the database, such as mechanical ventilation time, type of tracheostomy, laryngeal elevation, and tongue motility, which may have biased the results. Second, despite our attempts to control for confounders, there is still a risk of uncontrolled residual confounders, such as differences in tracheostomy management, consciousness level, or clinical interventions used. Third, the study was conducted at a single medical center with a unique clinical approach, and we did not have any information about discharges or other hospitals, which suggests the need for further validation to determine whether these results could be generalized to other hospitals or regions. In addition, this study focused only on the phases of swallowing and not on physiology, and the etiologies of pneumonia were not clarified. Whether the risk of pneumonia can be reduced by improving dysphagia in stroke patients with tracheostomy needs to be explored in future studies. Hence, the interpretation of these results should be undertaken with caution, and more high-quality prospective studies are urgently needed.

## 5. Conclusions

In summary, tracheostomy and dysphagia may increase the risk of pneumonia in patients with stroke. The total tracheostomy time, impaired or absent cough reflex, and oropharyngeal phase dysfunction were the risk factors for pneumonia, and there may be a correlation among PAS score, tracheostomy time, cough reflex, oropharyngeal phase dysfunction and UES opening. Patients with pneumonia had an increased hospital length of stay and higher costs. These findings suggest that appropriate management of tracheostomy combined with precise rehabilitation interventions for dysphagia may be considered to reduce the risk of pneumonia in patients with stroke, dysphagia and tracheostomy. Therefore, future prospective studies with larger sample sizes should be conducted to verify our findings. In addition, the relationship between tracheostomy and dysphagia remains unclear, and further studies are needed.

## Figures and Tables

**Table 1:** Operational definitions of different phases of swallowing.

Phases of Swallowing	Operational Definitions	Start Point	Endpoint
Oral preparatory phase	Before the swallowing reaction begins, the food is chewed and formed into a bolus in the oral cavity	The bolus was processed in the oral cavity	
Oral propulsive phase	The passage of food bolus from the oral cavity to the pharynx	The tongue voluntary presses the collected bolus against the palate	The head of the bolus reaches the intersection of the mandibular ramus and the base of the tongue
Pharyngeal phase	The bolus from the pharynx to the esophagus entrance	Bolus passing the ramus of the mandible	The upper esophageal sphincter relaxation
Esophageal phase	The bolus goes down the esophagus and into the stomach	The upper esophageal sphincter relaxation	The bolus into the stomach

**Table 2:** Clinical characters of tracheostomy patients with dysphagia after stroke.

Variables	Pneumonia Group (n = 140)	No Pneumonia Group (n = 40)	*p*-Value
Male, n (%)	100 (71.5)	31 (77.5)	0.447
Age (year), mean (SD) Stroke type, n (%) Hemorrhagic Ischemic Stroke site, n (%) Supratentorial Infratentorial Other	54.0 (15.7) 52 (37.1) 89 (62.9) 78 (55.7) 35 (25) 27 (19.3)	54.8 (14.2) 12 (30) 28 (70) 22 (55) 10 (25) 8 (20)	0.341 0.405 0.995
Feeding behavior, n (%)			
Oral feeding	27 (19.3)	9 (22.5)	0.654
Gastric tube feeding Intermittent intubation feeding Partial oral feeding	64 (45.7) 17 (12.2) 32 (22.8)	15 (37.5) 7 (17.5) 9 (22.5)	0.356 0.379 0.962
History of disease, n (%)			
Coronary Heart Disease	16 (11.4)	5 (12.5)	0.852
Hypertension	85 (60.7)	26 (65.0)	0.623
Diabetes	33 (23.5)	10 (25.0)	0.852
Hyperlipidemia	11 (7.8)	5 (12.5)	0.363
Smoker, n (%)	21 (15.0)	4 (10.0)	0.584
Drinker, n (%) Total tracheostomy times (month) Number of instances of ventilator support, mean (SD) Length of hospital stay (day) Direct medical costs (USD)	15 (10.7) 6.3 ± 5.9 0.41 (0.493) 36.0 ± 7.2 15,702.21 ± 14,244.61	3 (7.5) 4.3 ± 1.7 0.18 (0.385) 30.5 ± 11.7 10,923.99 ± 7250.14	0.765 0.037 * 0.007 * 0.025 * 0.042 *

Note: SD, standard deviation; USD, United States Dollar; * *p* < 0.05.

**Table 3:** Analysis of swallowing function on pneumonia.

Variables	Pneumonia Group	No Pneumonia Group	*p*-Value	Effect Size	95% CI
	n = 89	n = 27			
PAS, mean (SD) UES opening, n (%) Complete Not complete Cough reflex, n (%) Present Dysfunction (impaired/absent)	5.2 (1.92) 59 (66.3) 30 (33.7) 21 (23.6) 68 (76.4)	4.3 (1.79) 16 (59.3) 11 (40.7) 12 (44.4) 15 (55.6)	0.039 * 0.503 0.035 *	0.458 0.124 0.399	0.024 to 0.893 −0.24 to 0.489 0.028 to 0.770

Note: SD, standard deviation; UES, Upper Esophageal Sphincter; PAS, Penetration-Aspiration Scale; * *p* < 0.05. 95% CI, 95% Confidence Interval.

**Table 4:** Differences in the phase of swallowing between groups.

The Phase of Swallowing, n (%)	Pneumonia Group (n = 89)	No Pneumonia Group (n = 27)	*p* Value	Effect Size	95% CI
Oral phase Pharyngeal phase Esophageal phase Oral + Pharyngeal phase (Oropharyngeal) Pharyngeal + Esophageal phase Oral + Pharyngeal + Esophageal phase	8 (8.9) 18 (20.2) 0 54 (60.6) 4 (4.5) 5 (5.7)	3 (11.1) 9 (33.3) 0 10 (37.1) 2 (7.4) 3 (11.1)	0.742 0.158 0.031 * 0.918 0.580	0.061 0.264 0.408 0.019 0.102	−0.303 to 0.425 −0.102 to 0.631 0.037 to 0.780 −0.344 to 0.383 −0.261 to 0.467

Note: * *p* < 0.05. 95% CI, 95% Confidence Interval.

**Table 5:** Post hoc testing between groups.

The Phase of Swallowing, n (Adjusted Residual)	Pneumonia Group (n = 89)	No Pneumonia Group (n = 27)
Oral phase Pharyngeal phase Esophageal phase Oral + Pharyngeal phase (Oropharyngeal) Pharyngeal + Esophageal phase Oral + Pharyngeal + Esophageal phase	8 (−0.3) 18 (−1.4) 0 54 (2.2) * 4 (−0.6) 5 (−1)	3 (0.3) 9 (1.4) 0 10 (−2.2) 2 (0.6) 3 (1)

Note: Adjusted residuals appear in parentheses to the right of observed frequencies. * The difference is statistically significant.

**Table 6:** The multivariate logistic regression analysis for the risk factors of pneumonia.

Variables	OR	95% CI	*p*-Value
Total tracheostomy time	5.040	1.966 to 12.922	0.001 **
Cough reflex dysfunction	0.241	0.084 to 0.695	0.008 **
Present of oropharyngeal phase dysfunction	2.976	1.087 to 8.148	0.034 *

Note: OR, odds ratio; 95% CI, 95% Confidence interval; * *p* < 0.05; ** *p* < 0.01.

**Table 7:** The correlation analysis of tracheostomy and dysphagia.

	PAS Scores	UES Opening	Cough Reflex	Oropharyngeal Phase Dysfunction	Total Tracheostomy Time	Number of Instances of Ventilator Support
PAS scores	1	0.118	0.277 *	0.318 *	0.178 *	0.024
UES opening	0.118	1	0.139	0.643 **	−0.033	0.36
Cough reflex	0.277 *	0.139	1	0.549 **	−0.010	0.223
Oropharyngeal phase dysfunction	0.318 **	0.643 **	0.549 **	1	−0.010	0.034
Total tracheostomy time	0.178 *	−0.033	−0.010	0.59	1	0.016
Number of instances of ventilator support	0.024	0.36	0.223	0.034	0.016	1

Note: * *p* < 0.05; ** *p* < 0.01.

## Data Availability

The authors declare that data supporting the findings of this study are available within the article.
